# The Utility of FDG-PET/CT in Clinically Suspected Paraneoplastic Neurological Syndrome: A Literature Review and Retrospective Case Series

**DOI:** 10.3389/fneur.2017.00238

**Published:** 2017-06-01

**Authors:** Mark P. Maskery, Jonathan Hill, John R. Cain, Hedley C. A. Emsley

**Affiliations:** ^1^Department of Neurology, Royal Preston Hospital, Preston, United Kingdom; ^2^University of Manchester, Manchester, United Kingdom; ^3^Department of Nuclear Medicine and Imaging, Royal Preston Hospital, Preston, United Kingdom; ^4^Department of Radiology, Royal Preston Hospital, Preston, United Kingdom; ^5^Faculty of Health and Medicine, Lancaster University, Lancaster, United Kingdom

**Keywords:** neuroimmunology, paraneoplastic syndromes, neurooncology, FDG PET/CT, imaging techniques

## Abstract

Paraneoplastic neurological syndrome (PNS) describes a spectrum of rare, heterogeneous neurological conditions associated with an underlying malignancy. Diagnosis of PNS is inherently difficult, with frequent misdiagnosis and delay. The literature suggests an underlying immune-mediated pathophysiology, and patients are usually tested for the presence of onconeural antibodies. With direct tumor therapy being the most effective method of stabilizing patients, there is a strong emphasis on detecting underlying tumors. The sensitivity of conventional CT imaging is often inadequate in such patients. While FDG-PET imaging has already been shown to be effective at detecting these tumors, FDG-PET/CT, combining both structural and functional imaging in a single study, is a more recent technique. To study the utility of FDG-PET/CT, we conducted a systematic literature review and a retrospective study. We identified 41 patients who underwent imaging for clinically suspected PNS at the regional PET-CT and neurosciences center based at the Royal Preston Hospital between 2007 and 2014 and compared the results to conventional investigations. Five patients had FDG-PET/CT tracer avidity suspicious of malignant disease, and four of these were subsequently diagnosed with cancer. Sensitivity and specificity were calculated to be 100 and 97.3%, respectively, with positive predictive value 80% and negative predictive value 100%. This compares to a sensitivity and specificity of 50 and 100%, respectively, for CT and 50 and 89%, respectively, for onconeural antibodies. These findings are in line with previous studies and support the diagnostic accuracy of FDG-PET/CT for the detection of underlying malignancy.

## Introduction

Paraneoplastic neurological syndrome (PNS) is a rare presentation of an occult, underlying malignancy (1–6), which is often susceptible to misdiagnosis (7). It can affect the central nervous system, peripheral nervous system, and the neuromuscular junction (8) and this, in part, accounts for a variable constellation of clinical features. While PNS can present due to virtually all cancers (9), it is most prominently associated with small cell lung cancer (SCLC) but also commonly reported with other tumors such as breast, ovarian, thymic, and lymphoid (9–12). It develops in less than 1% of cancer patients (3, 5), and in contrast to the direct or metastatic effects of the tumor (13) it is widely regarded to be immune mediated (1, 7, 9, 14, 15). In 2004, an international panel of experts recommended criteria to aid clinicians in defining a neurological syndrome as paraneoplastic dependent on onconeural antibodies, the presence of an underlying malignancy, and categorizing presentation into “classical” and “non-classical” syndromes; with “classical” syndromes (e.g., Lambert-Eaton myaesthenic syndrome, limbic encephalitis, encephalomyelitis, subacute cerebellar degeneration, sensory neuronopathy, dermatomyositis, or opsoclonus-myoclonus) being more likely to be associated with an underlying malignancy (2). PNS is characterized by a rapidly progressive debilitating neurological disorder (1), which, in most patients, manifests before the malignancy becomes symptomatic (4, 7). The majority of underlying malignancy presents within 4–6 months, although the literature suggests an interval of up to 4 years (7, 16).

The immune-mediated pathophysiology leads to the production of onconeural antibodies (2, 7, 17). The concept of these antibodies is continuing to evolve and as yet they have an uncertain and diverse role in the pathogenesis of PNS (7). Such antibodies are widely regarded to prime the immune system against a mutual antigen, common to both neural tissue and underlying tumor (9), often causing irreversible neuronal damage (18). While their detection has been reported as useful to distinguish a presentation as paraneoplastic in origin (19), their use in neurological practice is inherently limited (2). Primarily, this is due to their presence in patients without PNS; alongside their frequent absence in patients in whom PNS is clinically suspected (9). The spectra of known onconeural antibodies continue to expand and hence we are unable to confidently rule out PNS with current antibody panels.

Treatment of PNS adopts three domains: direct tumor therapy, symptomatic management, and immunotherapy (8). Currently direct tumor therapy, in effect removing the underlying antigenic source, is seen as the most definitive method of treatment and therefore detection of underlying malignancy is of paramount importance to patient management (7, 15, 20).

The rarity of PNS inevitably means there is a paucity of information on which to base guidelines for diagnosis. In the past decade, there have been four key publications in this area (2, 4, 21, 22).

Overall, PNS is a rare and difficult diagnosis, and currently the most effective method of stabilizing the patient is direct tumor therapy. This relies on timely, accurate detection of an underlying malignancy and conventional modalities such as CT that lack the coregistration of metabolic activity provided by FDG-PET/CT imaging, are not always sufficiently sensitive (11, 23). Further complicating the situation is the possibility that, on occasion, tumors identified by imaging are incidental to the clinical presentation. While recent guidance does acknowledge the role of nuclear medicine in the diagnosis of selected patients (4, 5), we have evaluated the utility of this modality through a systematic review of the literature and a single center, retrospective study.

### Literature Review

A review of the available English-language literature was conducted to identify similar studies. While our retrospective study focusses entirely on the utility of ^18^F-fluoro-deoxyglucose positron emission tomography with low dose computed tomography for attenuation correction (FDG-PET/CT), conventional FDG-PET studies were also included for comparative purposes. MEDLINE (1946 to March 2016) was searched utilizing the following terms: “*Positron Emission Tomography*” and “*Paraneoplastic Syndromes, Nervous System*.” These were then searched using the following keywords: paraneoplastic neurologic* syndrome*, paraneoplastic neurologic* disorder*, paraneoplastic syndrome*, paraneoplastic disorder*, positron emission tomography, PET, FDG-PET, and *18F FDG-PET*. The relevance of each result was determined, and references were reviewed to identify missing studies. The studies that reported sensitivity and specificity are summarized in Table 1.

**Table 1:** Reported outcomes of similar studies.

Reference	Sample size	Description	Sensitivity (%)	Specificity (%)
**FDG-PET-imaging**				
Younes-Mhenni et al. (25)	20	Positive onconeural antibodies and previously negative conventional imaging	>83	25
Linke et al. (26)	13	Onconeural antibody-positive patients	90	67
Patel et al. (27)	104	Clinically diagnosed PNS	80	67
**GC-PET imaging**				
Hadjivassiliou et al. (1)	80	Clinically diagnosed PNS	75	87–92
**FDG-PET/CT imaging**				
Vaidyanathan et al. (13)	68	Clinically diagnosed PNS	100	82
Schramm et al. (3)	66	Clinically diagnosed PNS	100	91
Kristensen et al. (28)	67	Clinically diagnosed PNS	75	88.9

The detection of underlying malignancy using FDG-PET functional imaging has been acknowledged for more than a decade; however, due to the more recent emergence of combining structural and functional imaging in a single study with FDG-PET/CT technology, there is relatively less evidence for the use of this modality in PNS.

Five similar studies using FDG-PET imaging modality were identified (11, 24–27), which included between 13 and 104 patients and, where reported, demonstrated a sensitivity and specificity of 80–90 and 25–67%, respectively. However, in contrast to this current study, three authors included only those patients with positive onconeural antibody status (24–26).

One study (1) evaluated gamma camera positron emission tomography (GC-PET) with low-dose CT for attenuation correction in 80 patients and reported sensitivity and specificity of 75 and 87–92%, respectively. The authors concluded that when used early in the clinical course, FDG-PET imaging has the potential to reduce the utilization of further expensive investigations.

Six similar studies have analyzed the use of FDG-PET/CT in PNS (3, 10, 13, 20, 23, 28). These included between 27 and 68 patients and were mostly retrospective. In accordance with our own retrospective study, which aimed to evaluate diagnostic performance in everyday neurological practice, all the other studies included patients with clinically suspected PNS, regardless of onconeural antibody status. Sensitivity and specificity were reported in three of these, with sensitivity reported between 75 and 100% and specificity ranging between 82 and 91% (3, 13, 28). In contrast, none of the studies identified during the literature review developed and utilized a tool for FDG-PET/CT interpretation. These studies universally acknowledge the diagnostic value of FDG-PET/CT but are divided in opinion of how this modality should be utilized in clinical practice. Kristensen et al. (28) reported findings on a total of 137 patients; however, these included patients presenting with neurological (*n* = 67), rheumatological (*n* = 25), dermatological (*n* = 18), nephrological (*n* = 6), hematological (*n* = 2), abnormal biochemistry (*n* = 11), and others (*n* = 6), hence only the data for the 67 patients presenting with clinical suspicion of neurological disorder have been included.

## Materials and Methods

### Patient Selection

This retrospective study has been based entirely on preexisting patient records and imaging, which was acquired during routine clinical care. It was considered to constitute service evaluation and as such not to require ethical approval.

All FDG-PET/CT imaging requests between December 2007 and May 2014 at a regional PET-CT and neurosciences center were retrospectively analyzed to identify those carried out due to clinically suspected PNS. Patients were excluded from the study in the event that their case notes were unavailable or if the diagnosis of PNS had not been made by a consultant neurologist. All patients with clinically suspected PNS were intentionally included in order to reflect routine clinical practice.

Patient demographic information, clinical presentation, and previous imaging were recorded from the notes. Where onconeural antibodies were found to be positive, subtypes and titers were collected. Follow-up information, including the outcomes of subsequent imaging, biopsy, presence of malignancy, treatment and final diagnosis, was recorded. A final diagnosis of PNS was based on documentation of this diagnosis by the responsible clinician.

### FDG-PET/CT Protocol

All patients underwent FDG-PET/CT scan, with FDG acquisition from skull base to upper thigh with low-dose CT for attenuation correction. Patients were imaged according to a set protocol using a GE Discovery STE 64 slice PET-CT scanner. Non-diabetic patients were required to fast for 6 h prior to the administration of 300–400 mBq of ^18^F-FDG produced at the on-site cyclotron, aiming for preinjection blood glucose of 4–8 mmol/L. Diabetic patients were required to fast for 4 h prior to attending and continue insulin/metformin treatment prior to the administration of ^18^F-FDG, with a target preinjection random blood glucose <12 mmol/L. Following administration, patients were required to relax for 60 min in a dark, warm, comfortable “uptake” room before being scanned in order to minimize brown fat and muscle uptake (29).

### FDG-PET/CT Interpretation

Scans were reviewed by a Radiology registrar (ST4) with an interest in nuclear medicine, under the supervision of a consultant in radiology and nuclear medicine. According to FDG tracer pattern, they were assigned to one of five categories (see Table 2). Grade I scans exhibited physiological biodistribution of FDG uptake with no abnormality. Grade II scans were those which, while anomaly was detected, this was assumed to be a physiological variation. Grade III scans had anomalous tracer patterns but there was no definitive evidence of underlying malignancy. Grade IV and V scans showed tracer avidity in keeping with malignancy, with grade V being reserved for those scans demonstrating definitive or widespread malignant disease.

**Table 2:** Grading system for FDG PET/CT interpretation.

Category	Grade	FDG uptake interpretation
Negative exam	I	Normal
	II	Slight abnormality, presumed physiological
	III	Abnormality of uncertain significance
Positive exam	IV	Suspicious abnormality for underlying disease
	V	Definitive/widespread malignant disease

Grade IV and V scans were classified as a positive result, since there was no definitive evidence for malignancy in scans graded I–III.

For comparative purposes, in the event that prior conventional CT imaging had been undertaken, this prior scan was assigned to one of three categories according to interpretation of the radiology report. Grade I scans were considered normal, grade II scans were equivocal, and grade III scans were suspicious of malignancy. Grade I–II scans were considered negative, and grade III scans positive.

### Laboratory Investigation

Where patients had undergone investigation of certain onconeural antibodies, the outcome was recorded. Antibodies were selected according to current literature and through personal communication with a consultant immunologist at the hospital.

### Statistical Analysis

Sensitivity, specificity, positive predictive value (PPV), and negative predictive value (NPV) were calculated according to all available clinical evidence. Fisher’s exact test was carried out for differences in proportions of patients with malignancy identified by FDG-PET/CT and conventional CT imaging.

## Results

### Patient Demographics

Fifty-three patients were identified who underwent FDG-PET/CT for clinically suspected PNS. Ten external patients, in whom case notes were unavailable, were excluded. Two patients were excluded since they were not referred by a consultant neurologist. Forty-one patients were included, 17 males and 24 females. Mean age was 58 (range 18–83 years). One patient was included with a previously treated malignancy, not thought to be related to this subsequent referral.

Patients were followed up for an average of 3 years, 4 months. Patients with a negative FDG-PET/CT had an average follow-up time of 3 years, 6 months (range 7–2,302 days). The patient with a positive FDG-PET/CT in whom a malignancy had not been subsequently detected was followed up for 2 years, 11 months.

Twenty patients received a final diagnosis of PNS. Fourteen patients were diagnosed with a classical PNS; eight patients with subacute sensory neuronopathy, four patients with limbic encephalitis, one patient with encephalomyelitis, and one patient with subacute cerebellar degeneration. Six patients received a diagnosis of non-classical PNS, including two patients diagnosed with motor neuron disease and one patient with each of optic neuritis, stiff person syndrome, paraneoplastic myelopathy, and myasthenia gravis. The remaining 21 patients received an alternative final diagnosis, documented in Table 3.

**Table 3:** Final diagnoses in patients without confirmed paraneoplastic neurological syndrome.

Patient no.	FDG-PET/CT grade	CT grade (where available)	Onconeural antibody status	Final diagnosis
1	II		Negative	Sensory neuropathy
2	III	I	Negative	Sarcoidosis
4	I	I	Negative	Sjögrens syndrome
9	II		Negative	Complex partial seizures
16	IV		Negative	Mononeuritis multiplex
17	III	I	Negative	Myelopathy
20	III	II	Negative	Sarcoidosis
22	I	I	Negative	Cerebellar ataxia
23	I		Negative	Possible MS
25	I	II	Negative	Atypical parkinsonism
28	III		Negative	Peripheral polyneuropathy
31	I		Negative	Myelopathy
32	III		Negative	Sjögrens syndrome
36	I		Negative	Idiopathic late-onset cerebellar ataxia
38	III		Negative	Dry beriberi
39	II		Negative	Sensory neuropathy
41	III		Positive	Morvan’s syndrome
43	I	I	Negative	Inflammatory neuropathy
46	III	I	Negative	Subacute encephalopathy
49	I		Negative	Multilevel radiculopathy
50	II	I	Negative	Idiopathic sensory polyneuropathy

Follow-up identified four patients with underlying malignancy. Three patients had histological confirmation (two SCLCs, one endometrial cancer), and one patient who was deemed unfit for biopsy had both clinical and radiological evidence of thyroid malignancy and received palliative care. Of these four patients with an underlying malignancy, three presented with a classical PNS, and one with non-classical (see Table 4).

**Table 4:** Outcomes of patients with a positive FDG-PET/CT result.

Patient no.	Age at FDG-PET/CT	Gender	Clinical features	Onconeural antibody status	Previous imaging	FDG-PET/CT interpretation (grades I–V)	Evidence of malignancy	Clinical diagnosis
5	71	Female	Rapidly progressive neuropathy	Anti-Hu positive	CT negative	IV	Endometrial cancer	Subacute sensory neuronopathy
6	72	Male	Rapidly progressive dementia, right hemiparesis and dysphasia	Negative	CT negative	IV	Thyroid malignancy	Encephalomyelitis
16	77	Female	General muscle wasting, reduced left knee jerk, decreased function in left and intermittent arm swelling	Negative	No previous imaging	IV	Nil	Mononeuritis multiplex
29	51	Male	Poor balance, nystagmus, mild facial palsy, dysphagia^a^	Anti-Hu positive	CT positive	V	Small cell lung cancer (SCLC)	Subacute cerebellar degeneration
52	68	Male	Reduced sensation from the waist down, weakness in the legs	Negative	CT positive	IV	SCLC	Paraneoplastic myelopathy

*^a^Dysphagia in this patient was not thought to be due to PNS but due to an FDG avid lymph node identified on FDG-PET/CT, which appeared to encroach the esophagus*.

### Interpretation of Imaging

Thirteen FDG-PET/CT scans were interpreted as grade I, 6 scans as grade II, 17 as grade III, 4 as grade IV, and 1 as grade V. Hence, there were 5 positive FDG-PET/CT scans (outcomes of which are summarized in Table 4) and 36 considered negative. There was positive correlation between identification of underlying malignancy and positive FDG-PET/CT. The single false positive result, combined with negative onconeural antibody status, was later diagnosed with mononeuritis multiplex.

Of the 41 clinically suspected PNS patients, 5 FDG-PET/CT scans were considered positive. Malignancy was detected in four of these patients, and no patient having a negative FDG-PET/CT was found to have malignancy (*P* < 0.0001, Fisher’s exact test). Therefore, sensitivity and specificity is 100 and 97.3%, respectively, with PPV 80% and NPV 100%. As shown in Table 5, these findings are in line with previous studies.

**Table 5:** Three previous studies of the accuracy of FDG-PET/CT for detecting underlying malignancy have shown sensitivity and specificity and these are in line with our findings.

	No. of patients	Sensitivity (%)	Specificity (%)
Vaidyanathan et al. (13)	68	100	82
Schramm et al. (3)	66	100	91
Kristensen et al. (28)	67	75	88.9
Our findings	41	100	97.3

Prior CT was identified for 22 patients (54%), with a median time before FDG-PET/CT of 10.5 days. A total of 16 patients received CT imaging of the chest, abdomen, and thorax, 3 patients received thorax and abdomen, 2 patients had imaging of the chest only, and 1 patient received a CT head. Fifteen scans were normal, five were equivocal, and two scans were suspicious for malignancy. Therefore, 2 scans were positive and 19 were considered negative. Both positive scans were associated with an underlying malignancy (SCLC); however, the staging of this malignancy was more accurate with subsequent FDG-PET/CT. Two patients with a negative CT scan were subsequently found to have an underlying malignancy.

Conventional CT was undertaken in 22 patients prior to FDG-PET/CT and 2 were considered positive for malignancy (*P* = 0.0266, Fisher’s exact test). Both of these patients had an underlying malignancy detected. However, two patients with negative CT had a malignancy which was later detected with FDG-PET/CT. Sensitivity therefore is 50%, specificity 100%, PPV 100%, and NPV 90%.

### Onconeural Antibody Investigation

Forty patients underwent onconeural antibody testing. Anti-Hu, anti-Ma2, anti-Yo, anti-Ri, anti-CV2, and anti-amphiphysin were tested collectively as part of a standard serum panel. Two patients were found to be positive for anti-Hu antibodies. One anti-Hu-positive patient, presenting with subacute cerebellar degeneration, was found to have underlying SCLC and one patient, who presented with a subacute sensory neuronopathy, had endometrial cancer. Anti-AChR, anti-VGCC, anti-VGKC, and anti-NMDA receptor antibodies were tested in selected patients, and two results were returned positive for each of anti-VGKC and anti-NMDA receptor antibodies. While none of these were associated with underlying malignancy, 3 patients were diagnosed with limbic encephalitis and 1 patient, with a highest anti-VGKC antibody titer of 223 pM, was diagnosed with Morvan’s syndrome.

Overall, onconeural antibodies were investigated in 40 patients, with positive results returned in 6 of these. Of the four patients diagnosed with underlying malignancy, two had positive onconeural antibodies, and two were negative. Sensitivity and specificity, therefore, were 50 and 89%, respectively.

## Discussion

Follow-up identified 20 patients with a final clinical diagnosis of PNS, and 4 (20%) of these were found to have malignancy during follow-up, this is proportionally less than previously documented. While this could be attributed to the length of follow-up, it is likely to be a reflection on selection criteria. This study is based on routine neurological practice; therefore, patients were selected regardless of onconeural antibody status and upon suspicion, rather than formal diagnosis, of PNS.

The literature shows that accurate identification of an underlying malignancy in patients with clinically suspected PNS is critically important for stabilization of the neurological syndrome. Early identification of tumors such as SCLC could also allow for a more favorable patient outcome. The findings of this study are in line with previous similar studies (see Table 5) and show that FDG-PET/CT is a useful imaging modality in the detection of such malignancies.

Due to the potential for false negatives in both onconeural antibody investigation and conventional CT, the sensitivity of both of these investigations was 50%. Contrastingly, FDG-PET/CT had a sensitivity of 100% since it accurately detected all patients with malignancy. Indeed, 3 of the 14 patients with a classical PNS had an underlying malignancy, 2 of which were occult on CT scanning. However, this is at the expense of a slightly lower specificity than conventional CT, and the associated higher number of false positive results on FDG-PET/CT could lead to unnecessary clinical investigation. Furthermore, the relatively high cost of FDG-PET/CT often leads to CT scanning being the default first-line imaging modality for clinically suspected PNS, despite the possibility of additional radiation exposure in some patients, where negative CT is still followed by FDG-PET/CT.

**Figure 1:**
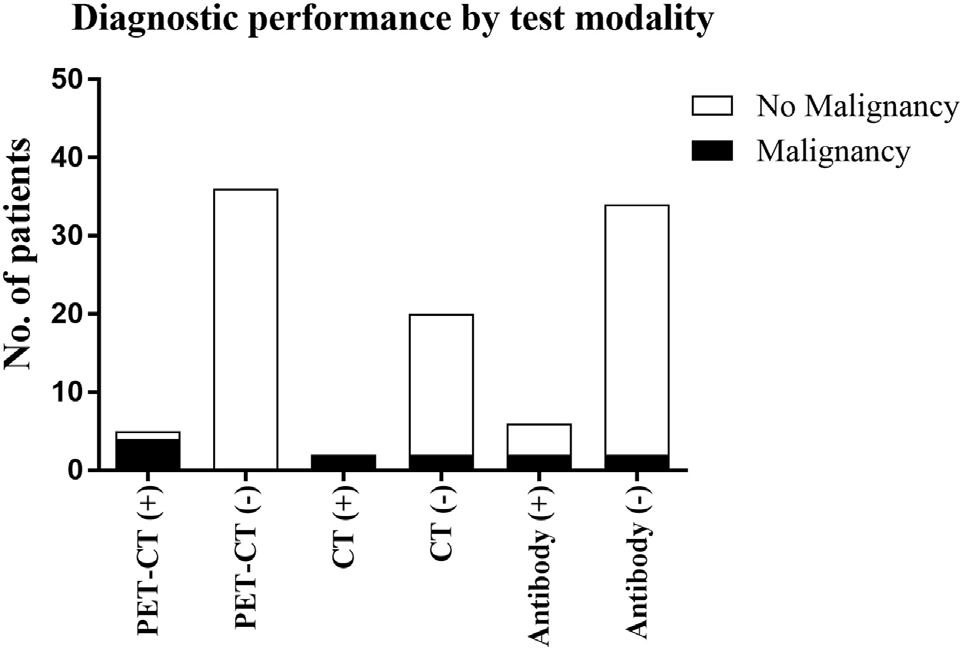
The outcomes of the three diagnostic techniques evaluated in this study and the detection of malignancy associated with these.

As summarized in Figure 1, our study demonstrates the diagnostic performance of FDG-PET/CT in a typical cohort of patients with clinically suspected PNS, and its utility should be considered in their clinical management. However, patient selection is ultimately the key to ensuring the appropriateness of CT or FDG-PET/CT as the best first-line imaging modality, factoring in diagnostic performance, cost and radiation exposure.

## Ethics Statement

This retrospective study, carried out at the Royal Preston Hospital, Preston, Lancashire, United Kingdom, has been based entirely on preexisting patient records and imaging which was acquired during routine clinical care. It was considered to constitute service evaluation and as such not to require ethical approval.

## Author Contributions

MM, JH, JC, and HE made a substantial contribution to the design, data collection, and analysis of the research and drafting of the manuscript and have reviewed and accepted the contents of the manuscript prior to submission. HE was the lead investigator for the study.

## Conflict of Interest Statement

The authors declare that the research was conducted in the absence of any commercial or financial relationships that could be construed as a potential conflict of interest.
